# Influence of RF Power on Structural and Corrosion Properties of Sputter‐Deposited CrN Films

**DOI:** 10.1002/jemt.70059

**Published:** 2025-08-14

**Authors:** Alireza Grayeli, Sahar Rezaee, Ştefan Ţălu

**Affiliations:** ^1^ Physics and Accelerators Research School Nuclear Science & Technology Research Institute Tehran Iran; ^2^ Department of Physics Ker. C., Islamic Azad University Kermanshah Iran; ^3^ The Directorate of Research, Development and Innovation Management (DMCDI) Technical University of Cluj‐Napoca Cluj‐Napoca Romania

**Keywords:** CrN films, Minkowski functional, potentiodynamic polarization, surface micromorphology, x‐ray diffraction

## Abstract

This study examines the deposition of chromium nitride (CrN) films on 304 stainless steel (304SS) substrates using radio‐frequency (RF) magnetron sputtering, with RF power varied from 20 to 160 W in an argon atmosphere. The structural, topographical, and corrosion resistance properties of the films were analyzed using X‐ray diffraction (XRD), atomic force microscopy (AFM), scanning electron microscopy (SEM), and electrochemical polarization measurements. Employing the atomic force microscope, morphological characteristics of CrN films under constant conditions using Minkowski Functionals were analyzed. Subsequently, by examining parameters like root‐mean‐square roughness, skewness, and kurtosis, the study revealed the variations in the particle distribution and their probability density as a function of deposition power alterations. Results indicate that increasing RF power enhances film crystallinity, as evidenced by intensified XRD peaks. With an increase in RF power reaching up to 80 W, there is a significant enhancement in the uniformity of the surface, resulting in an evenly spread grain pattern. Corrosion tests in 0.5 M sulfuric acid, assessed via potentiodynamic polarization, identify 80 W as the optimal RF power for maximizing corrosion resistance. This study provides insights into the relationship between film structure, surface characteristics, and corrosion behavior, highlighting key factors influencing protective performance.

## Graphical abstract

- Three‐dimensional AFM images of CrN/304SS films synthesized at varying RF power levels.
- Height distribution and the curves of the bearing area of CrN/304SS films synthesized at varying RF power levels.

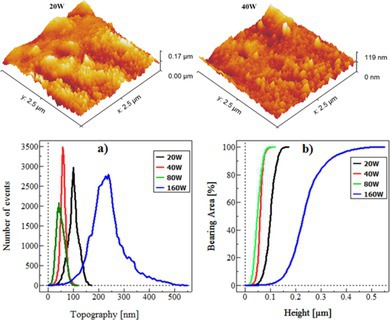

**Summary**

- RF power levels significantly improved CrN film crystallinity, with enhanced peak intensities and a preferential (111) orientation as power increased.
- Upon escalating the RF power to the threshold of 80 W, both a marked improvement in surface homogeneity and a pronounced uniformity of grains are evident, as confirmed by AFM imagery.
- Corrosion tests demonstrated optimal resistance at 80 W RF power; with further increases in power leading to reduced performance due to increased surface roughness.
- CrN films deposited at 160 W exhibited larger crystallite sizes but showed compromised corrosion resistance, highlighting the trade‐off between crystallinity and surface imperfections.
- This study emphasizes the importance of fine‐tuning RF power to balance crystallinity and surface smoothness for optimized film properties.

## 1 Introduction

Austenitic 304 stainless steel (304SS) has garnered substantial research interest due to its extensive utilization in environments where corrosion resistance is a critical requirement. This alloy, primarily composed of iron with a minimum of 17% chromium, exhibits an intrinsic capacity to develop a self‐protective oxide layer upon exposure to atmospheric oxygen and moisture. This passive film serves as an effective barrier, mitigating further electrochemical degradation of the underlying material and enhancing its corrosion resistance (Aslam et al. 2022; Zaffor et al. 2021; Shakoury et al. 2019). The formation of this protective oxide layer occurs in both gaseous and aqueous environments, where it manifests as a thin yet compact film that significantly reduces the corrosion rate. However, the stability and durability of this passive layer are highly dependent on various extrinsic and intrinsic factors, including the duration of exposure, environmental conditions, temperature variations, and the specific chemical composition of the alloy (Das et al. 2020; Wang et al. 2020).

While the corrosion performance of austenitic stainless steels is predominantly dictated by their inherent chemical composition, researchers have increasingly explored surface engineering techniques as a means of further augmenting their resistance to corrosive degradation. Several advanced surface modification strategies have been extensively investigated, including gas and ion nitriding, ion implantation, and physical vapor deposition (PVD). Among these, PVD‐based processes have gained considerable traction due to their environmentally sustainable characteristics and ability to produce coatings with superior mechanical and corrosion‐resistant properties (Sadeghi et al. 2023; Vaca et al. 2021; Babur et al. 2021; Habibi et al. 2022; Huang et al. 2019; Karimi and Grayeli 2019). In particular, transition metal nitrides have emerged as highly promising protective coatings due to their exceptional hardness, wear resistance, and chemical stability. Furthermore, extensive research has been dedicated to optimizing the microstructural and phase composition of stainless steel substrates through the incorporation of additional alloying elements. Nickel and chromium, for instance, have been the subject of numerous investigations due to their profound influence on corrosion resistance, significantly enhancing the passivation behavior and structural integrity of the material (Okonkwo et al. 2022; D'Souza et al. 2021; Guo et al. 2020).

Metal carbides and nitrides have increasingly attracted attention within the field of materials science, primarily owing to their distinctive physicochemical properties, including high melting points, superior corrosion resistance, and unique electrical and magnetic characteristics. These attributes render them viable candidates for replacing noble metals in a diverse range of industrial applications (Lei et al. 2008; Peri et al. 2022). Additionally, investigations into the oxidation behavior of transition metal carbides and nitrides have revealed distinct differences in their activation energy requirements, with carbides typically exhibiting lower oxidation activation energies than their nitride counterparts. This distinction underscores the need for comprehensive studies on their thermal stability and oxidation kinetics to further optimize their functional performance in harsh operating conditions (Lim et al. 2020).

Among transition metal nitrides, chromium nitride (CrN) has been widely recognized for its outstanding combination of mechanical, tribological, and corrosion‐resistant properties, as well as its remarkable thermal stability. These characteristics make CrN films highly versatile for a broad spectrum of industrial applications, including wear‐resistant coatings for cutting tools, protective layers in medical and food processing equipment, automotive components, and microelectronic devices. The resilience of CrN coatings enables them to withstand extreme environmental conditions, including elevated temperatures and aggressive chemical exposure. Investigations by Ruden‐Muñoz et al. (Ruden‐Munoz et al. 2015) examined the tribological and mechanical performance of CrN coatings deposited on steel substrates via magnetron sputtering, revealing that films synthesized at lower pressures (0.4 Pa) exhibited superior adhesion properties. Similarly, Shah (Shah 2017) explored the influence of partial nitrogen pressure and gaseous composition on the microstructural evolution of CrN coatings, highlighting the crucial role of deposition parameters in determining film characteristics. In a complementary study, Liu et al. (Liu et al. 2003) evaluated the thermal stability of CrN films, reporting a gradual decline in hardness from 21 GPa at room temperature (298 K) to 10 GPa at 773 K for a 2 mm‐thick coating, thereby demonstrating the temperature‐dependent mechanical behavior of these films. Furthermore, research conducted by Zalnezhad et al. (Zalnezhad et al. 2013) highlighted the effectiveness of CrN coatings on Al7075‐T6, demonstrating that their high hardness, corrosion resistance, and mechanical stability make them ideal for tool protection. Using varying DC power, temperature, and nitrogen flow rates, they measured surface hardness via microhardness testing and developed a fuzzy logic model that predicted coating hardness with 94.664% accuracy.

Various PVD techniques have been employed for the deposition of CrN coatings, including magnetron sputtering (Grudinin et al. 2022) and vacuum arc discharge (Ovcharenko et al. 2015), among others. Of these methods, magnetron sputtering has been widely adopted due to its ability to achieve high deposition rates, uniform film thickness, and minimal thermal impact on the underlying substrate.

Subramanian and Jayachandran (Subramanian and Jayachandran 2011) conducted electrochemical analyses on ~2 μm thick CrN coatings deposited by DC reactive magnetron sputtering on low‐carbon steel. XPS confirmed the presence of CrN with minor Cr_2_O_3_ content, while XRD revealed (111) and (200) crystalline orientations. AFM showed continuous rectangular cell‐like surface patterns. Corrosion testing in 3.5% NaCl using potentiodynamic polarization indicated improved performance, confirming the CrN film's effectiveness as a corrosion‐resistant barrier. Merie et al. (Merie et al. 2016) studied CrN thin films deposited on silicon substrates via reactive magnetron sputtering, with constant deposition parameters except for substrate temperature (RT, 300°C, 500°C). AFM analysis showed that substrate temperature strongly affected surface morphology, roughness, and tribological behavior. Their results emphasize substrate temperature as a key factor in optimizing CrN film performance.

This research is dedicated to advancing the corrosion resistance of 304 stainless steel through the deposition of CrN coatings, with a primary focus on elucidating the influence of radio‐frequency (RF) power during magnetron sputtering. By systematically varying RF power, this study aims to optimize the microstructural characteristics and mechanical properties of CrN films to achieve superior corrosion protection. The precise control of RF power is hypothesized to govern key aspects of film crystallinity, adhesion strength, and surface morphology, thereby directly impacting the long‐term durability of the coated substrates in corrosive environments. The findings of this investigation are expected to contribute valuable insights into the optimization of PVD coating processes, facilitating the development of enhanced protective coatings for industrial applications where stainless steel components are subjected to aggressive operational conditions.

## 2 Experimental Details

### 2.1 Sample Preparation

CrN thin films were deposited onto 304SS substrates using the RF magnetron sputtering technique, with the substrate temperature meticulously maintained at 400°C to ensure optimal adhesion and film growth characteristics. The deposition parameters utilized in this process are detailed in Table 1.

**TABLE 1:** Deposition parameters for RF‐sputtered CrN films.

Substrate	304 stainless steel
Target	CrN
Substrate temperature [°C]	400
Target‐to‐substrate [mm]	120
RF power [W]	20, 40, 80, and 160
Base pressure [mbar]	1.5 × 10^−6^
Deposition pressure [mbar]	2 × 10^−3^
Deposition time [min]	60

**FIGURE 1:**
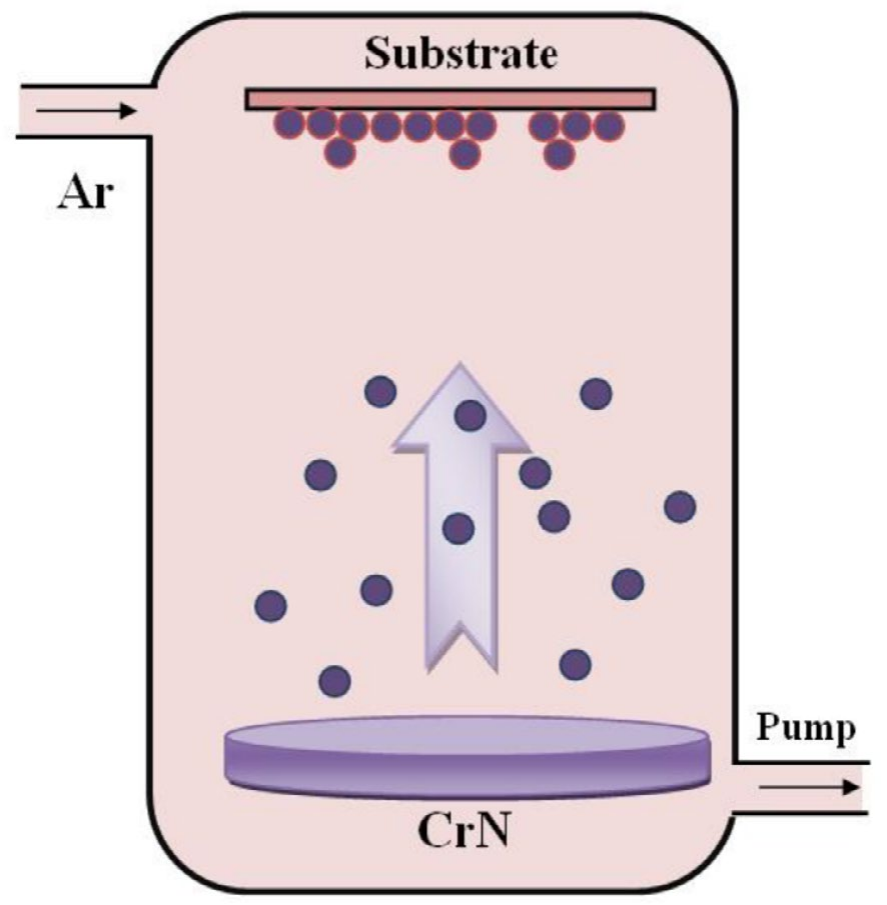
Schematic diagram of the RF sputtering system used for CrN films deposition.

The RF sputtering method, a widely recognized physical vapor deposition (PVD) technique, operates on the principle of plasma‐assisted material transfer, wherein high‐energy ions generated within the sputtering chamber bombard a chromium nitride target. This ion bombardment induces the ejection of CrN atoms and molecular clusters from the target surface, which subsequently condense and nucleate on the 304SS substrate, leading to the formation of a continuous, adherent thin film. The experimental setup employed for RF magnetron sputtering in this study is schematically illustrated in Figure 1.

To ensure precise control over the film deposition process, a high‐sensitivity quartz crystal deposition rate controller (Sigma Instruments, SQM‐160, USA) was strategically positioned in close proximity to the substrate. This instrument continuously monitored the deposition rate in real time, allowing for accurate assessment of material flux and ensuring uniform film thickness across the substrate surface. The precise monitoring of deposition rates is crucial for maintaining consistency in film properties, as variations in deposition rate can influence microstructural evolution, crystallinity, and surface morphology.

The chemical composition of the 304SS substrates employed in this study is shown in Table 2, detailing the elemental constituents and their respective weight percentages. The presence of chromium (≥ 17%) within the alloy composition is particularly noteworthy, as it plays a pivotal role in the formation of a passive oxide layer that enhances the intrinsic corrosion resistance of the stainless steel.

**TABLE 2:** Elemental composition of the 304SS substrate.

SS	Fe	Cr	Ni	Mn	Si	C	P	S
304	66–71 (%)	18–20 (%)	8–10.5 (%)	2 (%)	1 (%)	0.08 (%)	0.045 (%)	0.03 (%)

Prior to the deposition process, rigorous substrate preparation procedures were implemented to eliminate potential contaminants that could interfere with film adhesion and compromise the integrity of the deposited CrN layer. Initially, the protective polyethylene covering, which had been applied to the stainless steel sheets to prevent mechanical damage and oxidation during storage, was carefully removed. The substrates were then precisely cut to the required dimensions using a precision cutting tool to ensure uniformity across all samples. Following this, a comprehensive two‐stage cleaning protocol was executed to eliminate organic residues, surface oxides, and particulates.

In the first stage, the substrates were immersed in ethanol to dissolve and remove any residual polymeric material from the protective covering. Subsequently, an ultrasonic cleaning treatment was conducted in a heated acetone solution to effectively dislodge microscopic contaminants, including grease, dust, and residual hydrocarbons. This ultrasonic agitation process facilitated the penetration of the solvent into surface irregularities, ensuring a thorough cleansing effect. Following the acetone treatment, the substrates underwent a final rinse with ethanol to eliminate any remaining solvent residues. Immediately after this cleaning sequence, the substrates were dried in a controlled environment to prevent airborne contamination before being transferred into the sputtering chamber for film deposition.

To establish a contamination‐free environment conducive to high‐quality thin‐film deposition, the sputtering chamber was evacuated to a high vacuum level, achieving a base pressure of less than 1.5 × 10^−6^ mbar. This rigorous evacuation process effectively eliminated residual gases and impurities, thereby minimizing potential contamination that could compromise film integrity. A high‐purity CrN target, with a diameter of 2 in. and an exceptional purity of 99.99%, was selected as the sputtering source to ensure the deposition of chemically consistent and structurally uniform films. The 304SS substrates were positioned at a fixed target‐to‐substrate distance of 120 mm, a critical parameter that influences the deposition rate, energy distribution of incoming species, and resultant film morphology. To further enhance the reproducibility and purity of the deposited films, a pre‐sputtering step was implemented prior to each deposition cycle. This procedure involved subjecting the CrN target to a 20‐min plasma etching process in an argon (Ar) atmosphere, effectively removing any residual surface contaminants, native oxides, or adsorbed species that could otherwise deteriorate the film's adhesion and microstructural homogeneity. The deposition process was conducted under a pure argon atmosphere, carefully maintaining a working pressure of 2 × 10^−3^ mbar to facilitate stable plasma generation and uniform material transfer. Chromium nitride films were deposited at four distinct radio‐frequency (RF) power levels—20, 40, 80, and 160 W—with each deposition being sustained for a fixed duration of 60 min.

The variation in RF power settings was intentionally selected to examine its impact on film growth kinetics, crystallinity, surface morphology, and corrosion resistance, aiming to optimize deposition conditions for superior protective performance of CrN coatings on stainless steel substrates.

### 2.2 Sample Characterization

The crystalline structure of the deposited CrN films was meticulously analyzed using X‐ray diffraction (XRD), performed with a STOE STADI MP diffractometer. The instrument was operated with CuKα radiation to ensure high‐resolution diffraction patterns, employing a step size of 0.01° and a counting time of 1.0 s per step to achieve precise peak identification and phase determination. This characterization facilitated the assessment of crystallographic orientations, phase composition, and the degree of crystallinity in response to varying RF power levels.

Atomic force microscopy (AFM) was performed in contact mode using an Auto Probe PC system (Park Scientific Instruments, USA). A low‐stress silicon nitride cantilever was employed, featuring a nominal tip radius of < 200 Å and a tip opening angle of 18°, optimized for high‐resolution surface imaging. The cantilever had an elastic (spring) constant of approximately 0.06 N/m and a resonance frequency of ~18 kHz, suitable for stable contact imaging without damaging the sample surface. The scan rate was maintained at 1 Hz to ensure accurate topographical data acquisition without introducing distortion due to tip dragging. Each image was captured with a resolution of 512 × 512 pixels, and multiple scan areas (2.5 × 2.5 μm^2^) were recorded to assess both global and localized surface features. All AFM measurements were conducted under ambient laboratory conditions, with a temperature of 22°C ± 1°C and relative humidity maintained at 50%, ensuring environmental stability during surface characterization. This technique provided critical insights into surface uniformity, grain size evolution, and morphological variations induced by different sputtering conditions.

The electrochemical properties of the CrN‐coated samples were systematically evaluated using the potentiodynamic polarization technique, employing a 273A potentiostat (EG&G, USA) interfaced with a computer for real‐time data acquisition and analysis. To precisely assess corrosion resistance, a small exposed surface area of approximately 1.0 cm^2^ was subjected to an acidic environment (0.5 M sulfuric acid solution). A three‐electrode electrochemical cell was utilized, consisting of a saturated calomel electrode (SCE) as the reference electrode, a platinum counter electrode, and the CrN‐coated sample as the working electrode. The polarization scan was executed at a controlled sweep rate of 1.0 mV·s^−1^, commencing from −400 mV relative to the open‐circuit potential (OCP). All electrochemical potential values reported in this study were referenced against the SCE, ensuring consistency in corrosion behavior interpretation.

The mechanical hardness of the CrN films was determined using a Vickers microhardness tester (Leitz Hardness Tester), applying a load of 25 g (equivalent to a force of 25.254 × 10^−4^ N) to the sample surface. This microindentation technique provided quantitative measurements of the films' resistance to deformation, facilitating comparative analysis of hardness variations as a function of RF power settings.

### 2.3 Statistical Analysis

Statistical analysis was performed using Origin Pro 2016 software, employing one‐way ANOVA with statistical significance set at *p* < 0.05.

## 3 Results and Discussion

### 3.1 Crystalline Structure Analysis via X‐Ray Diffraction (XRD)

**FIGURE 2:**
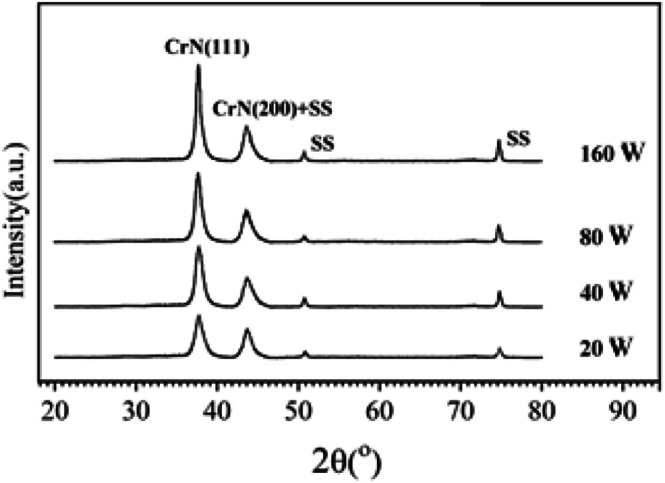
XRD patterns of CrN films deposited on 304SS substrates at varying RF power levels.

Figure 2 shows the X‐ray diffraction (XRD) patterns of CrN films synthesized at varying RF power levels, offering a comprehensive analysis of the relationship between RF power and the crystallinity of the films.

The XRD patterns demonstrate a clear trend of increasing phase intensity and the development of distinct diffraction peaks as the RF power is increased. For the film deposited at 20 W, the XRD pattern is predominantly dominated by peaks associated with the substrate, specifically the austenite phases of 304SS. The diffraction peaks at 2θ values of 43.71°, 50.78°, and 74.81° correspond to the γ‐Fe(111), γ‐Fe(200), and γ‐Fe(220) planes, respectively, which are characteristic of the underlying stainless steel. These observations indicate a relatively low degree of crystallinity in the CrN film at 20 W, as the CrN phase is not distinctly identifiable at this power level.

However, as the RF power is incrementally increased, the CrN phase (JCPDS 21‐1272) progressively crystallizes, with a pronounced preference for the (111) orientation. This preferred crystallographic orientation is consistently observed across all RF power settings from 40 W to 160 W, as evidenced by the distinct diffraction peak associated with the CrN(111) plane. Furthermore, a notable diffraction peak corresponding to the CrN(200) orientation appears at approximately 43.70°, which partially overlaps with the Fe(111) peak, indicating that the CrN phase continues to develop and crystallize as the RF power increases.

The degree of crystallinity in the films shows a clear enhancement with increasing RF power, evidenced by the growing intensity and sharpness of the diffraction peaks. This indicates that higher RF power promotes the ordering of the crystal structure, leading to a more well‐defined and ordered arrangement of CrN crystallites. Additionally, the observed increase in crystallite size can be attributed to the enhanced energy of the sputtered atoms, which increases as the RF power is raised. The higher energy of the sputtered atoms results from more intense ion bombardment during the deposition process, which increases the kinetic energy of the ejected particles. This higher energy facilitates the migration of atoms on the substrate surface, promoting nucleation and crystallization, ultimately leading to larger and more ordered crystallites with a stronger preference for the (111) orientation. Such an effect is well documented in the literature, where an increase in sputtering power is generally correlated with improved crystallinity and crystal size due to enhanced atomic mobility during deposition (Yan and Rath 2011; Koseoglu et al. 2015).

The crystallite size (*D*) was quantitatively determined using the Scherrer equation, which relates the diffraction peak broadening to the crystallite size (Nogueira et al. 2021):

(1) $$D=\frac{\mathrm{k\lambda}}{B\cos\theta }$$

where *k* is a dimensionless constant dependent on the crystallite morphology and distribution, *λ* is the wavelength of the X‐rays, θ is the Bragg angle, and *B* is the full width at half maximum (FWHM) of the diffraction peak (Çeşmeli and Biray Avci 2019). The FWHM value, *B*, was determined using the following relation:

(2) $$B=\sqrt{W_{0}^{2}-W_{i}^{2}}$$

where *W*_0_ is the FWHM of the sample under investigation, and *W*_i_ is the FWHM value of a reference sample, which is assumed to be free from stress.

Table 3 compiles the crystallite size values calculated using the Scherrer equation for the CrN(111) peak, along with surface roughness, microhardness, and corrosion parameters of the samples deposited at various RF power levels. A clear trend is observed: as the RF power increases, the crystallite size significantly increases, reflecting the enhanced film crystallinity and the growth of larger CrN crystallites. This is indicative of the elevated kinetic energy imparted to the sputtered species, which facilitates the growth of more ordered and larger crystallites.

**TABLE 3:** Crystallite size, hardness, and corrosion parameters of CrN films synthesized at different RF power levels.

Sample	RF power [W]		*D*_XRD_ [nm]	Microhardness [Hv]	Corrosion potential [V vs. SCE]	Corrosion current density [μA·cm^−2^]
304SS	—		—	508 ± 10	−0.23	90.18
CrN/304SS	1	20	24.52	2031 ± 18	0.26	5.20
	2	40	29.91	2312 ± 21	0.27	1.66
	3	80	38.74	2486 ± 45	0.30	0.71
	4	160	51.20	2501 ± 12	0.20	19.84

Abbreviation: *D*_XRD_, crystallite size.

These findings underscore the critical role of RF power in influencing the crystallographic and microstructural characteristics of CrN films. The increase in crystallinity and crystallite size is directly related to the improved protective performance of the CrN films, as the more ordered structures are expected to exhibit superior mechanical and corrosion resistance.

### 3.2 Surface Topography and Morphological Characterization by AFM

**FIGURE 3:**
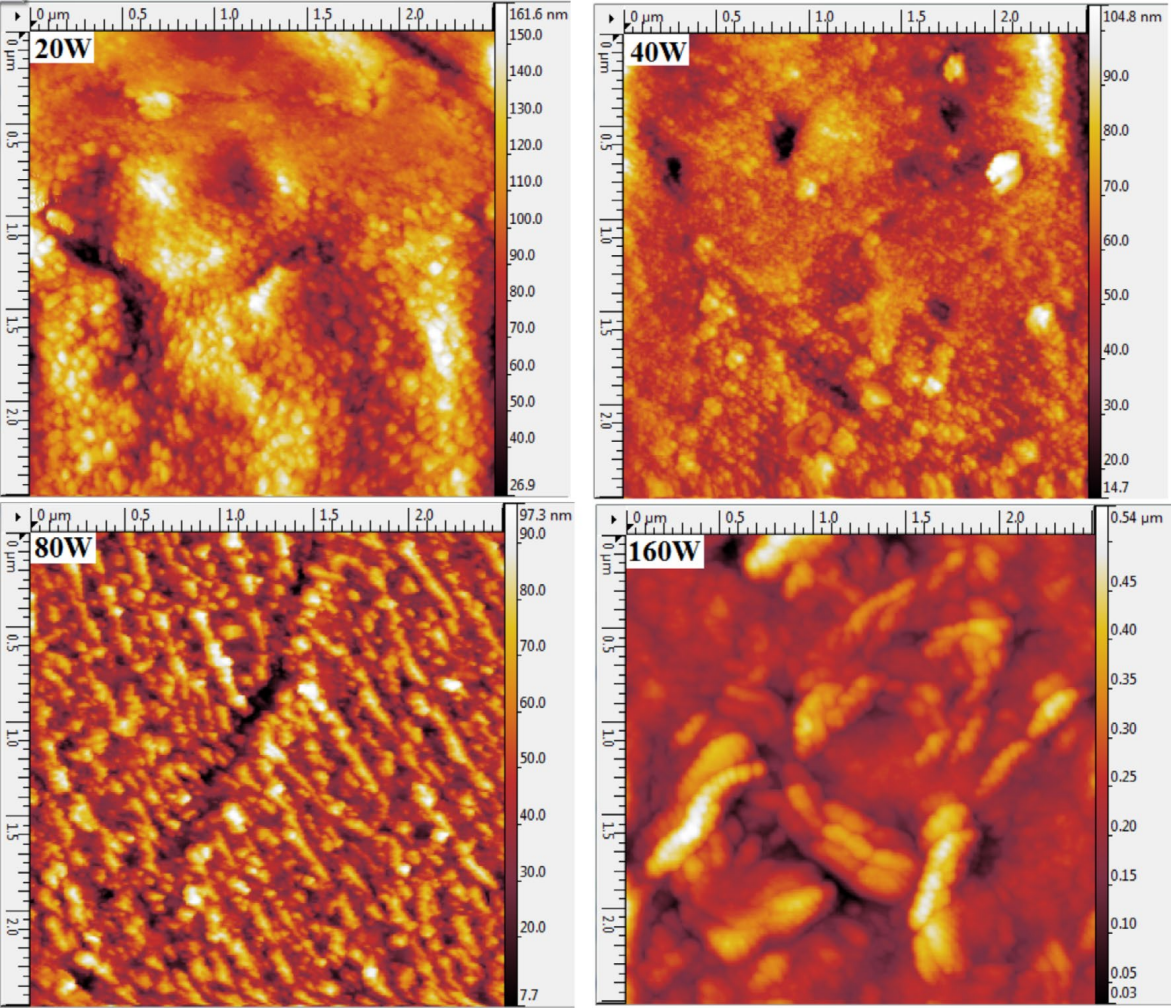
Two‐dimensional AFM images of CrN/304SS films synthesized at varying RF power levels.

The surface morphologies of the CrN films deposited at varying RF powers are depicted in Figure 3.

The two‐dimensional AFM images reveal a discernible evolution in the film's surface characteristics as a function of RF power. As the RF power increases up to 80 W, a notable improvement in surface homogeneity is observed, with a more uniform distribution of grains. The films produced at lower RF powers, particularly at 20 W, exhibit a relatively uneven and irregular surface structure. This irregularity can be attributed to the lower energy imparted to the sputtered particles, which results in non‐uniform growth and poor film adhesion.

**FIGURE 4:**
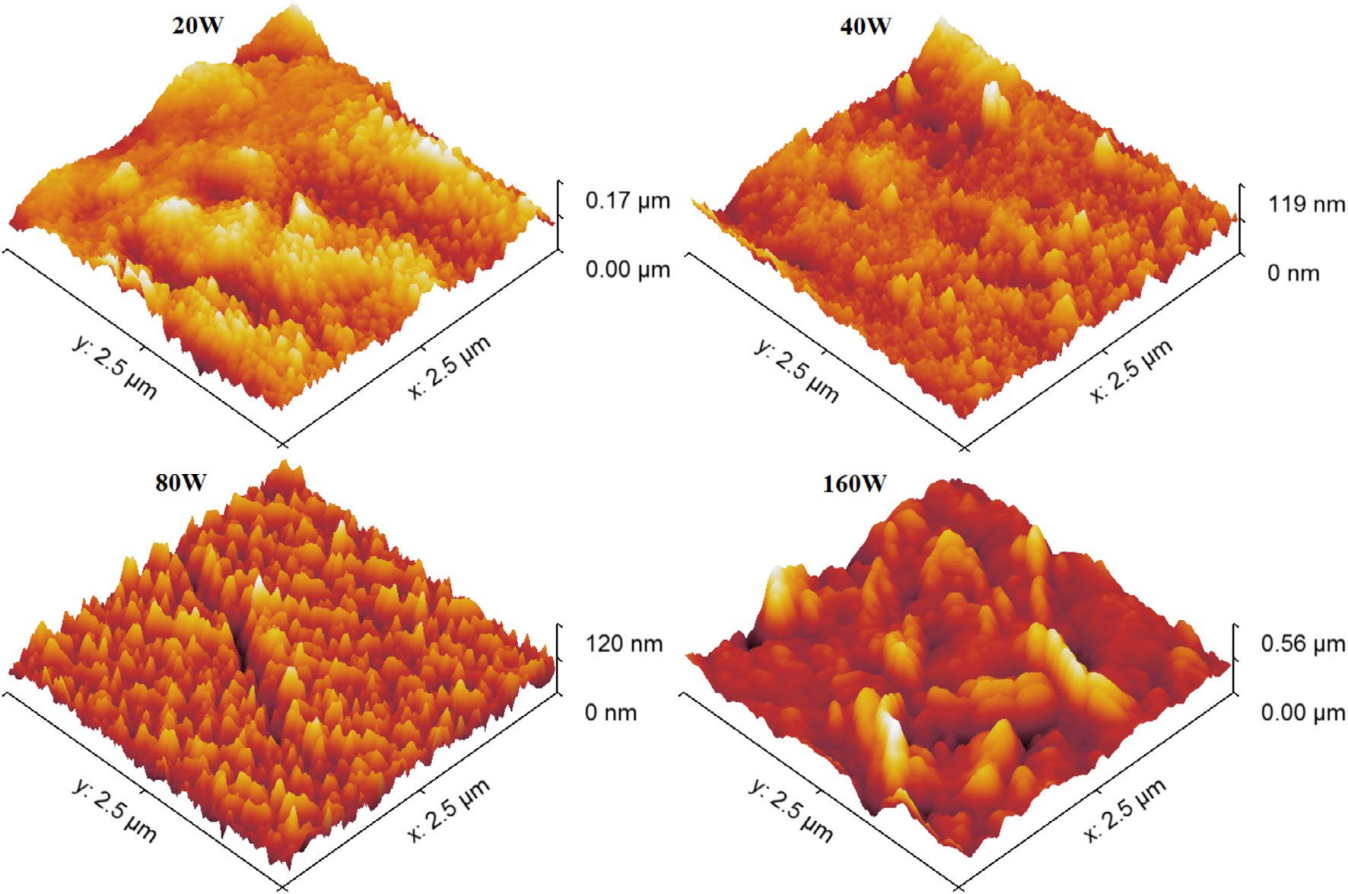
Three‐dimensional AFM images of CrN/304SS films synthesized at varying RF power levels.

As the RF power increases, the energy of the sputtered particles also increases, leading to a more uniform grain distribution. This energy enhancement facilitates the movement and coalescence of particles, resulting in a smoother and more homogeneous surface morphology. By 80 W, a surface with uniformly distributed grains is achieved, demonstrating the positive impact of increased RF power on the uniformity of film deposition. However, further escalation of the RF power to 160 W induces a different surface characteristic, wherein smaller grains coalesce to form larger, more defined grains, creating valleys between them, as shown in Figure 4. This phenomenon contributes to a significant increase in surface roughness, as evidenced by the increase in both average roughness (*R*_ave_) and root mean square roughness (Rms) values.

The increase in surface roughness observed at higher RF power levels can be linked to the coalescence of grains, which fosters the formation of these valleys. From a corrosion standpoint, these valleys facilitate a larger surface area for interaction with corrosive environments, potentially exacerbating material degradation, as will be discussed in the subsequent corrosion analysis. Thus, while higher RF power can improve grain uniformity up to a certain point, it also induces microstructural changes that may compromise the film's protective efficacy in corrosive conditions.

The relationship between RF power and surface morphology can be further elucidated through the Theory of Charged Clusters (TCC), which postulates that the aggregation and charging processes of sputtered atoms in the gas phase significantly influence the final surface structure (Ţălu 2015). As RF power increases, the number of free electrons in the system also increases, which in turn increases the likelihood of cluster formation and charging. This leads to the formation of clusters with larger sizes at higher RF powers, which, upon deposition, results in films with enhanced structural uniformity and a more ordered arrangement of grains (Eidsvåg et al. 2021; Sobola et al. 2017). The AFM images (both 2D and 3D) shown in Figures 3 and 4 offer compelling evidence of the strong correlation between RF power and surface morphology, emphasizing the significant role of energy dynamics in determining the final surface characteristics of the CrN films.

The CrN films exhibited a wide range of grain sizes, with a clear trend emerging from the data: films deposited at lower RF powers demonstrated notably smaller grain sizes, as evidenced in Figure 3. This observation is in line with the expected microstructural characteristics of CrN films under varying deposition conditions. The deposition process involves high‐energy particles that, upon collision with both the target and the substrate, undergo scattering, thereby significantly influencing the overall morphology of the films. These scattering events lead to variations in the packing density and distribution of grains within the films.

To comprehensively evaluate the influence of RF power on the surface morphology of the deposited films, atomic force microscopy (AFM) images were analyzed. Table 4 presents a thorough statistical analysis of the surface topography of CrN films deposited on 304 stainless steel (304SS) substrates under varying RF power levels (20–160 W). The evolution of surface morphology is quantitatively assessed using key parameters derived from atomic force microscopy (AFM), including height‐related metrics (*S*_a_, *S*_q_, *S*_p_, *S*_v_, *S*_z_), surface slope (*S*_dq_), asymmetry (*S*_sk_), peakedness (*S*_ku_), and fractal dimension (*D*_f_). The fractal dimensions were calculated using the cube counting method, employing linear interpolation to enhance the accuracy of the measurements and capture the complexity of the surface structures. These parameters collectively offer critical insights into the structural complexity and roughness behavior induced by changes in deposition energy.

**TABLE 4:** The statistical parameters of the surface samples of CrN films synthesized at different RF power levels.

The basic parameters	CrN/304SS samples			
	1	2	3	4
(*S*_a_) [μm]	0.016 ± 0.002	0.008 ± 0.001	0.012 ± 0.002	0.048 ± 0.004
(*S*_q_) [μm]	0.021 ± 0.003	0.011 ± 0.001	0.015 ± 0.002	0.065 ± 0.004
(*S*_p_) [μm]	0.073 ± 0.005	0.059 ± 0.004	0.070 ± 0.005	0.335 ± 0.011
(*S*_v_) [μm]	0.100 ± 0.006	0.060 ± 0.004	0.049 ± 0.004	0.285 ± 0.010
(*S*_z_) [μm]	0.174 ± 0.007	0.119 ± 0.006	0.120 ± 0.006	0.621 ± 0.012
(*S*_dq_) [°]	0.746 ± 0.003	0.530 ± 0.003	0.947 ± 0.004	2.455 ± 0.09
Skew (*S*_sk_)	−0.130 ± 0.005	0.127 ± 0.005	0.265 ± 0.007	0.487 ± 0.009
Kurtosis (*S*_ku_)	0.355 ± 0.007	1.773 ± 0.011	−0.258 ± 0.007	1.556 ± 0.011
*D*_f_	2.33 ± 0.01	2.34 ± 0.01	2.48 ± 0.01	2.28 ± 0.01

*Note*: arithmetic mean height (*S*_a_), root mean square height (*S*_q_), maximum peak height (*S*_p_), maximum pit depth (*S*_v_), maximum height (*S*_z_), skewness (*S*_sk_), kurtosis (*S*_ku_), surface slope (*S*_dq_), fractal dimensions (*D*_f_).

The arithmetic mean height (*S*_a_), representing the average surface deviation from the mean plane, exhibits a non‐linear response to RF power variations. At 20 W (Sample 1), *S*_a_ is 0.016 ± 0.002 μm, indicating a relatively smooth surface. This value decreases to 0.008 ± 0.001 μm at 40 W (Sample 2), suggesting densification or smoother growth. However, a moderate increase to 0.012 ± 0.002 μm at 80 W (Sample 3) precedes a substantial increase to 0.048 ± 0.004 μm at 160 W (Sample 4). The latter reflects enhanced surface roughening likely due to increased adatom mobility and re‐sputtering effects at higher power levels.

A parallel trend is observed in the root mean square height (*S*_q_), a more sensitive measure of surface roughness incorporating larger deviations. *S*_q_ decreases from 0.021 ± 0.003 μm at 20 W to 0.011 ± 0.001 μm at 40 W, then increases progressively to 0.015 ± 0.002 μm at 80 W and peaks at 0.065 ± 0.004 μm at 160 W. The concurrent increase in maximum peak height (*S*_p_) and maximum pit depth (*S*_v_), especially at 160 W (*S*_p_ = 0.335 ± 0.011 μm, *S*_v_ = 0.285 ± 0.010 μm), underscores the emergence of prominent topographical features, reflecting more aggressive growth modes and possible columnar or nodular morphology at high RF powers.

The maximum height (*S*_z_), as the total vertical distance between the highest peak and the deepest valley, also follows this trend. Its increase from 0.174 ± 0.007 μm (20 W) to 0.621 ± 0.012 μm (160 W) signals a significant broadening of the height distribution range, consistent with a rougher, more heterogeneous surface at increased powers.

The surface slope (*S*_dq_) provides additional information about the angular features of the surface. It exhibits a pronounced increase from 0.746° ± 0.003° (20 W) to 2.455° ± 0.09° (160 W); indicating a transition toward steeper and more inclined surface features. This steepening suggests increased microstructural anisotropy or the development of sharper features, likely tied to kinetic energy transfer during film growth at higher power settings.

Skewness (*S*_sk_) values shift from negative at 20 W (−0.130 ± 0.005), indicating a prevalence of valley‐like features, to increasingly positive values at higher powers, reaching 0.487 ± 0.009 at 160 W. This progression suggests a morphological transition from valley‐dominated to peak‐dominated surfaces, aligning with enhanced roughening and increased feature prominence.

Kurtosis (*S*_ku_), indicative of surface peakedness or flatness, shows a complex evolution. While Sample 2 (40 W) and Sample 4 (160 W) show increased kurtosis values (1.773 ± 0.011 and 1.556 ± 0.011, respectively), Samples 1 and 3 show lower or negative *S*_ku_ values (0.355 and −0.258), pointing to intermittent transitions between flatter and more pronounced surface topographies. These fluctuations likely reflect competition between smooth growth kinetics and surface instability effects, such as shadowing or roughening instabilities.

Previous studies have provided critical insights into the correlation between fractal parameters, surface roughness, and the optical properties of thin‐film materials, highlighting the pivotal role of scaling laws and surface complexity in determining the functional performance of nanostructured coatings (Pathak et al. 2025; Kumar et al. 2024; Vinita et al. 2024b; Țălu et al. 2017).

The values remain within a narrow range (2.28–2.48), yet reveal meaningful distinctions. A peak *D*_f_ of 2.48 ± 0.01 at 80 W implies a higher degree of surface intricacy or heterogeneity at this intermediate power, possibly due to the interplay between deposition rate and surface diffusion. Conversely, a lower *D*_f_ of 2.28 ± 0.01 at 160 W may suggest a coarsening effect or aggregation of surface features into larger, less intricate structures.

The observed morphological transformations with increasing RF power suggest that deposition energy critically governs surface roughness, texture, and complexity. At low to moderate powers (20–80 W), smoother and more homogenous surfaces are favored, potentially enhancing corrosion resistance or mechanical uniformity. At higher powers (160 W), surface features become more rugged, pronounced, and anisotropic, which may influence tribological performance and wear resistance in functional coatings. The complementary behavior of statistical parameters and fractal analysis underscores the multifaceted influence of RF power on thin film surface characteristics, enabling targeted design of CrN coatings with tailored properties.

**FIGURE 5:**
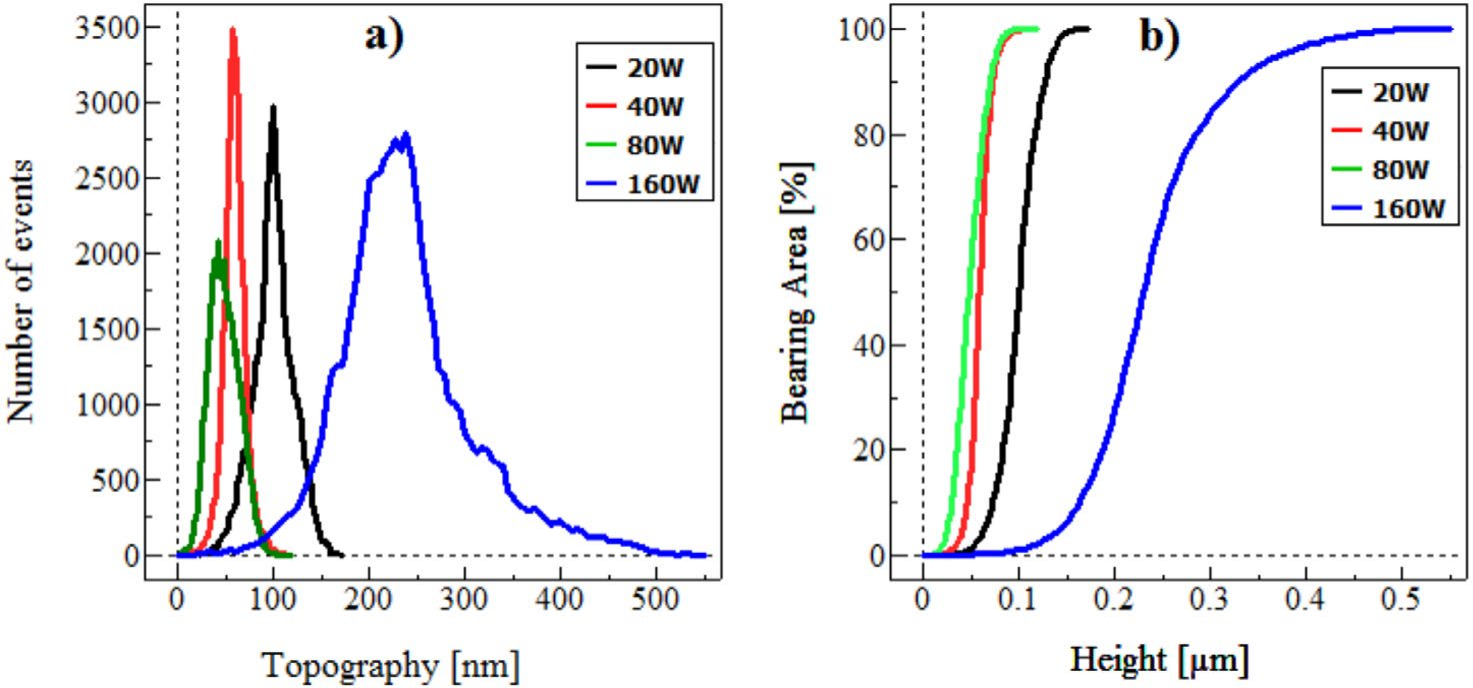
(a) Height distribution and (b) the curves of the bearing area of CrN/304SS films synthesized at varying RF power levels.

The correlation between Figure 5a and Tables 3 and 4 highlights that the Gaussian distribution's narrow width in particle height data can be indicative of smoother surfaces on films deposited at 40 W and 80 W, respectively; a fact supported by references (Noorbakhsh et al. 2023; Ahmadpourian et al. 2016). The Bearing Area Curve (BAC) (Bastami et al. 2025), which is represented in Figure 5b, is a critical statistical tool derived from surface profile measurements that provides insight into the load‐bearing capacity and functional performance of a surface. It quantifies the cumulative distribution of surface heights and is particularly useful in assessing the tribological and contact properties of thin films and coatings.

#### 3.2.1 Minkowski Functional

Minkowski functionals (MFs) (Grayeli et al. 2019) were applied to analyze the surface structure of CrN films on SS substrates at varied Rf powers. These MFs were computed using specific formulas in Gwyddion 2.59 software (Gwyddion Software n.d.; Vinita et al. 2024a).

The Minkowski volume (*V*) is calculated as $V\left(h\right)=1/2\left[\mathrm{erf}z\left(x,y\right)/1.414\mathrm{Sa}\right]$, while the Minkowski boundary (*S*) is given by Sh=k8πexp−zx,y22Sa, and the Minkowski connectivity (χ) is defined as χh=k2zx,y2π3Saexpzx,y22Ra.

**FIGURE 6:**
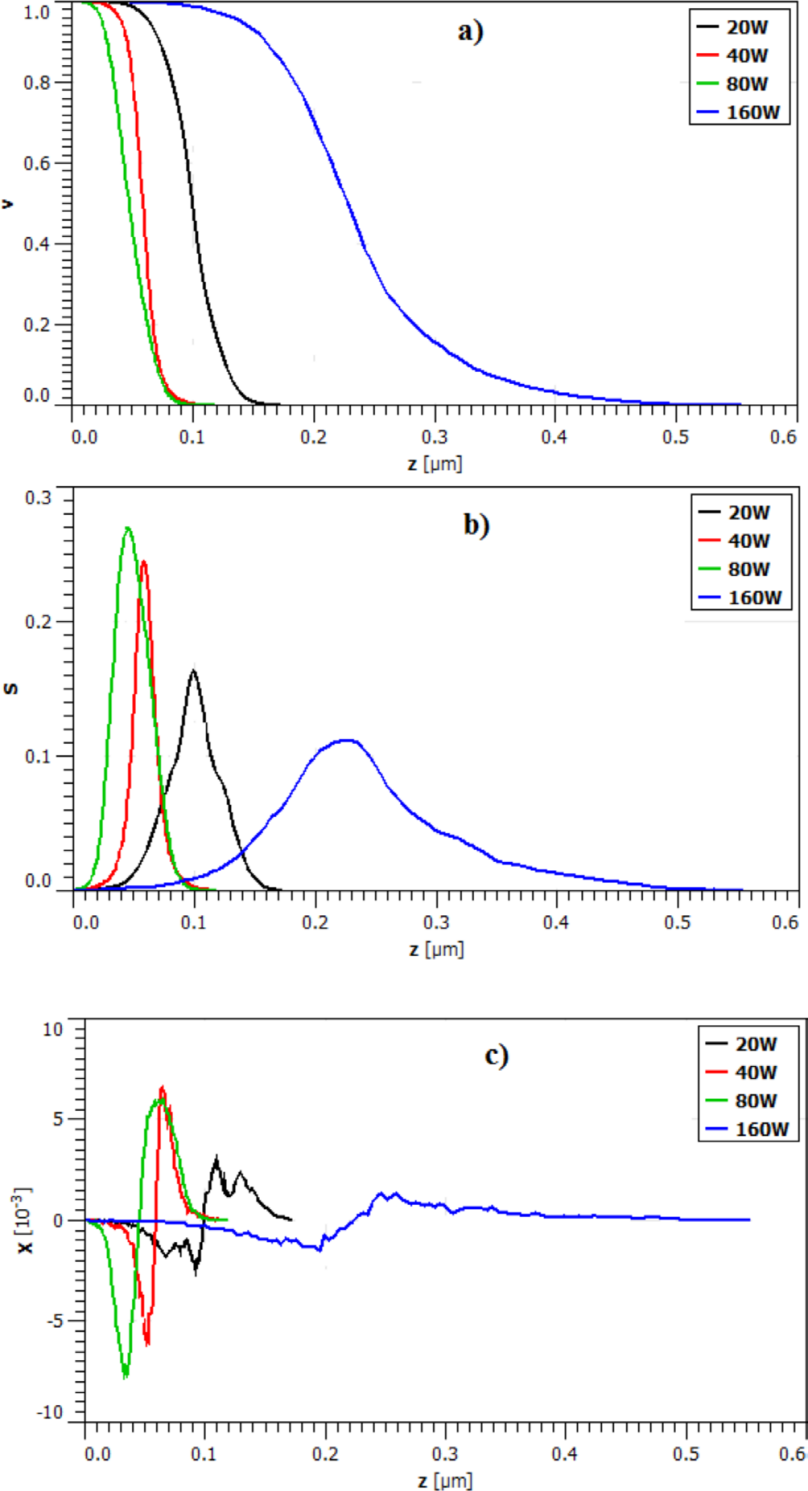
(a) Minkowski volume, (b) Minkowski boundary, and (c) Minkowski connectivity, respectively of CrN/304SS films synthesized at varying RF power levels.

These MFs have illuminated previously inadequate surface patterns. The complete surface pattern is depicted at *z* = 0 (Figure 6a), and the peak in normalized Minkowski volume (*V*) is identified.

As the *Z* value increases, the trend in the Minkowski volume diagram decreases, indicating slower changes in *V* for the sample deposited at 160 W compared to other samples. The Minkowski boundary (*S*) diagram for the samples mirrors the behavior seen in Figure 5a, with the diagram's position adjusted to the left. The Minkowski connectivity (*χ*), which relates to material conductivity on the surface and offers deeper insights into topology, exhibits a typical pattern with a peak and trough.

For the samples deposited at 40 and 80 W, the Minkowski connectivity (*χ*) shows two symmetric peaks with inflection points at *z* ≈0.0585, *z* ≈0.0488, and a range for the maximum and minimum points from 0.00 < *z* < 0.1083 and 0.00 < *z* < 0.1195, respectively (Figure 6c).

Additionally, for samples 40 and 80 W, the conductivity weakens as z approaches 0.1160. In contrast, for the 160 W sample, as *z* nears 0.5540, the intensity of the unidentified and asymmetrical peaks in the positive part of *χ*(*z*) decreases, suggesting a highly interconnected structure, aligning with the outcomes in Table 4 (*S*_q_, *S*_sk_, *S*_ku_, and *S*_z_).

### 3.3 Microhardness Characterization of Samples

The mechanical properties of CrN/304SS films synthesized at varying RF powers were systematically assessed, with a primary focus on hardness, a key parameter that reflects the material's resistance to deformation and wear. To obtain precise measurements, it was crucial to ensure that the indentation size was significantly smaller than the thickness of the CrN film, preventing any potential influence from the underlying substrate on the results. This approach minimized the impact of the substrate's hardness and allowed for an accurate representation of the CrN film's intrinsic hardness. Consistent experimental conditions were maintained across all samples, with a standardized load of 25 g applied during indentation, thereby ensuring the uniformity and reliability of the hardness comparisons across the different power levels.

To determine the microhardness of the CrN films, a Vickers microhardness tester was employed. The hardness values were calculated using the standard formula for Vickers hardness (Equation 3), where *l*_v_ denotes the length of the diagonal of the indentation and *F* represents the applied force:

(3) $$H_{v}=2\;\cos22^{o}\frac{F}{l_{v}^{2}}=1.854\frac{F}{l_{v}^{2}}\mathrm{kg}\;{\mathrm{mm}}^{-2}$$

This equation allows for the determination of the hardness by considering the size of the rhombus‐shaped indentation formed under the applied load. The formula provides a reliable method for quantifying the mechanical properties of the films, specifically their resistance to localized plastic deformation.

**FIGURE 7:**
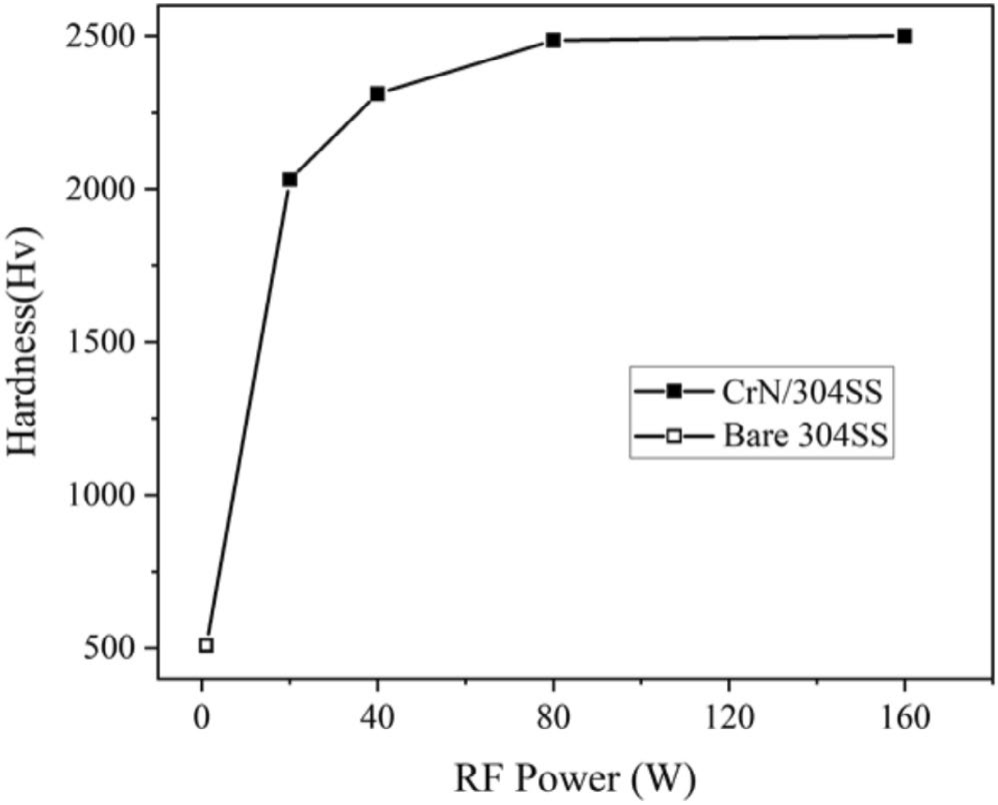
Hardness variation of CrN/304SS films as a function of RF power.

The results demonstrated a clear dependence of hardness on the RF power used during deposition. As shown in Figure 7, the CrN film deposited at 160 W exhibited the highest hardness, which can be attributed to its superior crystallinity and phase composition.

The corresponding X‐ray diffraction (XRD) analysis (Figure 2) indicates that the CrN(111) diffraction peak at 160 W is more intense than those observed at lower RF powers, suggesting that the film synthesized at this power exhibits the highest volume fraction of the CrN phase. This increased phase concentration contributes to the enhanced hardness observed in the 160 W deposited film. In contrast, the CrN film deposited at 80 W displayed a slightly lower hardness, though still significantly higher than that of the film deposited at 20 W. The increased hardness at 80 W is linked to a relatively high concentration of densely packed CrN phases, as evidenced by the strong CrN(111) and CrN(200) diffraction peaks in the XRD patterns (Figure 2). On the other hand, the CrN film deposited at 20 W showed the lowest hardness, which correlates with its lower crystallinity and reduced volume fraction of the CrN phase. This decrease in phase content at lower RF power is consistent with the observed morphological and structural changes in the films. Consequently, the hardness of the CrN films is strongly influenced by the RF power during deposition, with higher RF powers favoring a more crystalline structure and a higher content of the CrN phase, resulting in improved mechanical properties.

### 3.4 Corrosion Resistance of Samples

**FIGURE 8:**
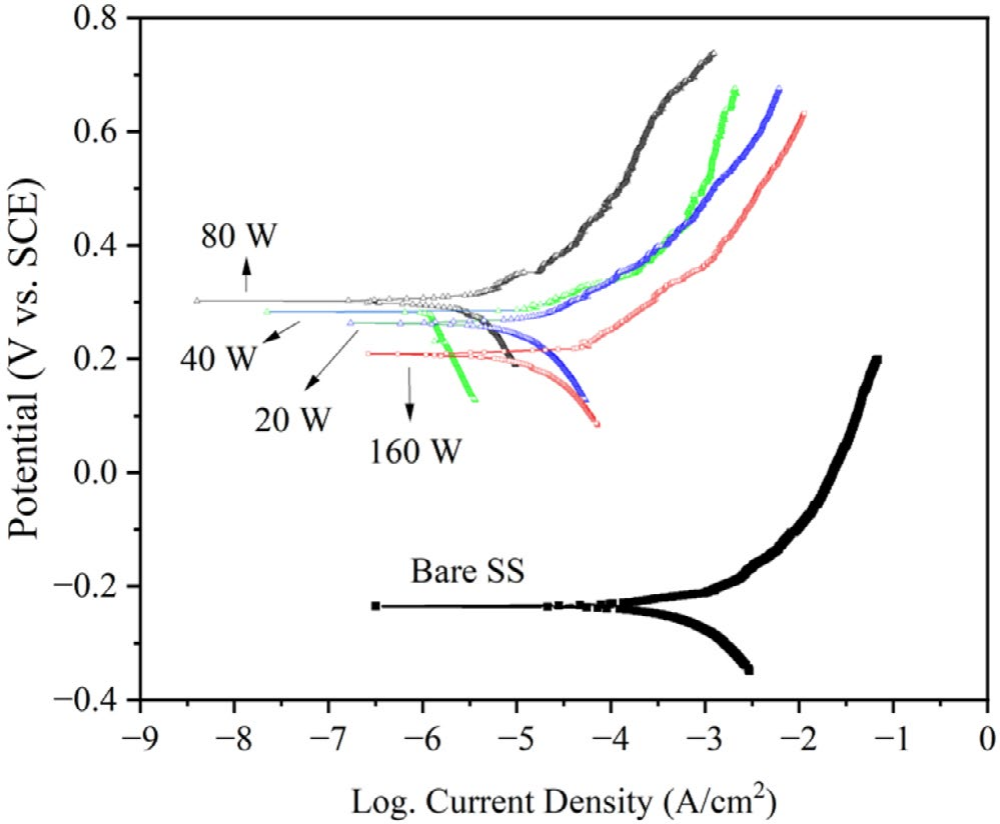
Polarization curves for bare substrate and CrN/304SS films synthesized at various RF powers.

The potentiodynamic polarization curves shown in Figure 8 provide valuable insights into the corrosion behavior of bare 304SS substrates, both uncoated and coated with CrN, in a 0.5 M H_2_SO_4_ solution across various RF power levels.

Notably, none of the samples exhibited classic active‐passive behavior, which is typically characterized by a clear shift from active dissolution to passive film formation at higher potentials. The corrosion resistance of the CrN coatings exhibited a clear dependency on the RF power, with a notable improvement in performance as the RF power was increased up to 80 W. In contrast, a decline in corrosion resistance was observed for the CrN film deposited at the highest power level (160 W), which can be attributed to an increase in surface roughness that facilitates the interaction of the coating with the corrosive solution.

The corrosion‐related parameters extracted from the polarization curves are summarized in Table 3, revealing a distinct trend: the corrosion resistance improves progressively with increasing RF power up to 80 W. This improvement is evidenced by an increase in the corrosion potential and a corresponding decrease in the corrosion current density. The CrN coating deposited at 80 W exhibited the most favorable corrosion performance, with a corrosion potential of 0.30 V and a corrosion current density of 0.71 μA cm^−2^. These values suggest that this specific power level leads to the most effective barrier against corrosion, offering enhanced protection for the underlying 304 SS substrate. In comparison, the uncoated stainless steel exhibited significantly higher corrosion rates, further highlighting the superior performance of the CrN coatings, particularly at 80 W RF power.

**FIGURE 9:**
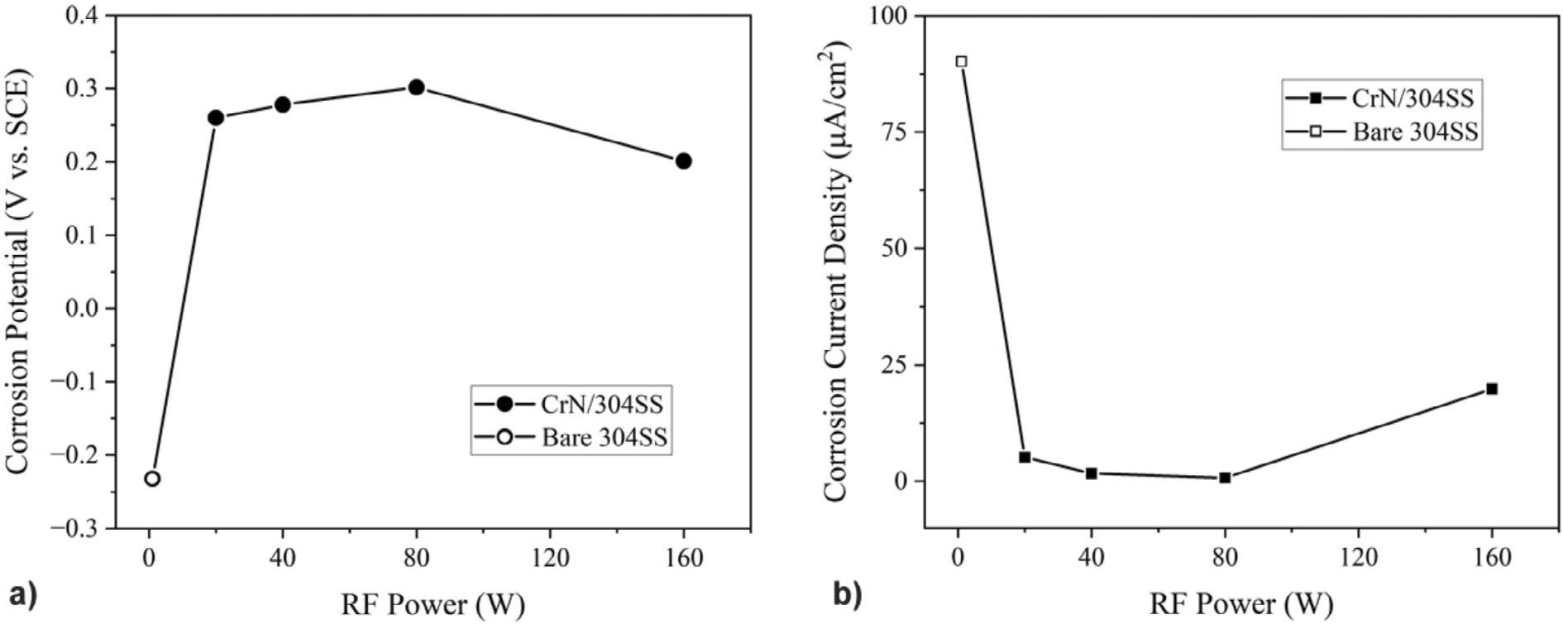
Dependence of (a) corrosion potential and (b) corrosion current density on RF power for CrN‐coated 304SS films.

The trends in corrosion resistance are clearly depicted in Figure 9a,b, which show the variations in corrosion potential and corrosion current density as a function of RF power.

By correlating the polarization data with the surface roughness measurements (Table 4), it becomes evident that a smoother surface topography plays a crucial role in enhancing the corrosion resistance of CrN coatings. This observation is in agreement with established research, which underscores the significant influence of surface morphology on the electrochemical behavior of coated materials (Rezaee et al. 2020). The results suggest that the optimization of RF power can be employed not only to improve the structural integrity and hardness of CrN films but also to fine‐tune their corrosion resistance properties for a wide range of applications.

### 3.5 SEM Analysis of Samples

The use of SEM enables detailed surface and morphological characterization at the micro‐ to nanoscale, as demonstrated in previous studies (Sobola et al. 2020; Knápek et al. 2019; Hoseinzadeh et al. 2018; Solaymani et al. 2018).

**FIGURE 10:**
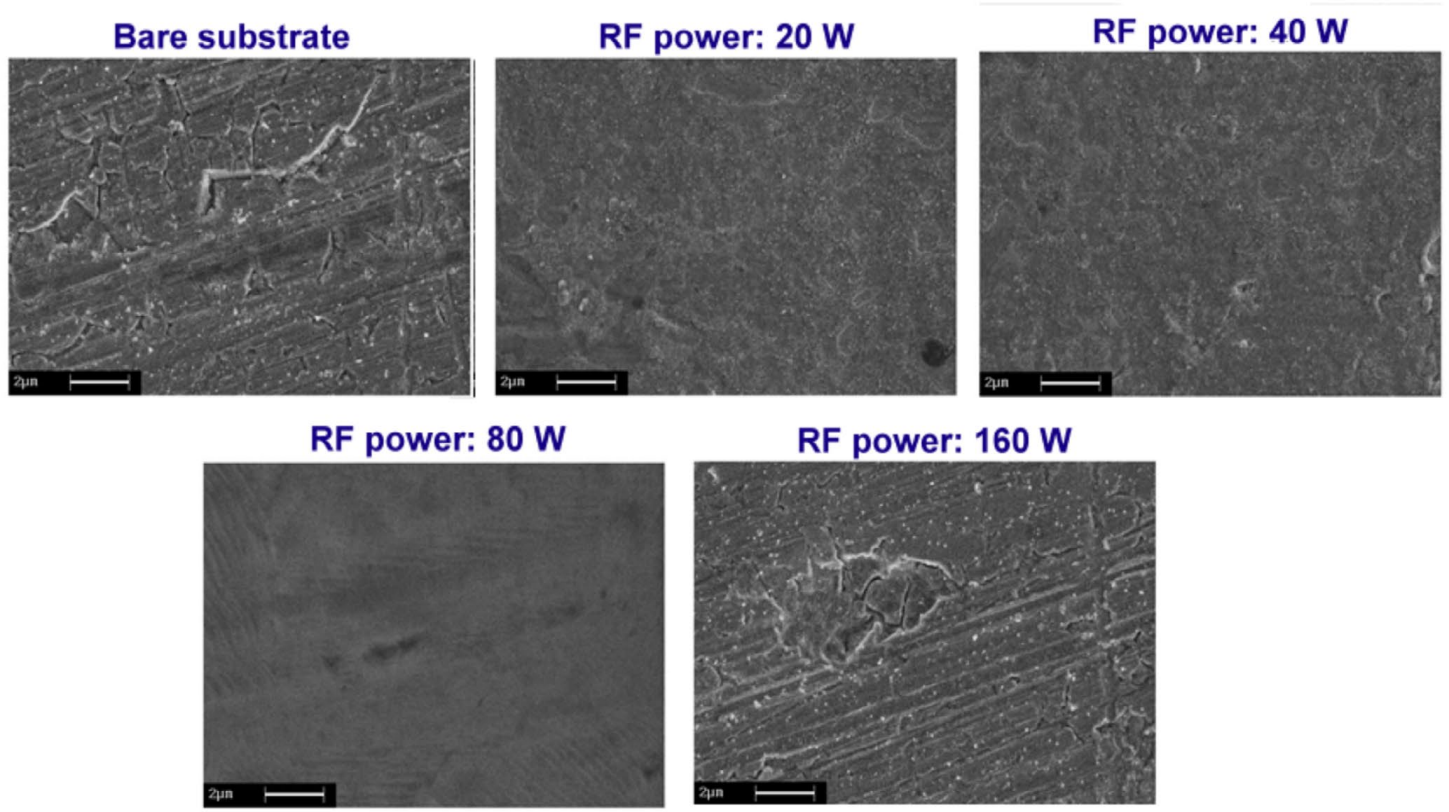
SEM images of the bare substrate and CrN/304SS synthesized at different RF powers.

Figure 10 shows SEM images of samples following corrosion testing, with particular emphasis on those coated at 80 W RF power, which exhibit significantly reduced surface damage compared to the other samples.

For comparative evaluation, Figure 10a includes a post‐corrosion image of an uncoated 304SS substrate, serving as a reference baseline to contextualize the protective efficacy of CrN coatings.

SEM analysis elucidates that CrN films synthesized at an RF power of 80 W manifest significantly enhanced corrosion resistance, evidenced by the near‐complete absence of macroscopic crack formation and surface degradation. This improvement is inherently linked to the minimization of structural discontinuities—particularly microcracks—which are commonly nucleated at surface heterogeneities and grain boundaries, subsequently facilitating the ingress of corrosive species. According to the Griffith fracture criterion, microstructural flaws such as cracks act as stress concentrators that reduce the effective fracture toughness of the film, thereby accelerating localized corrosion mechanisms. In contrast, CrN‐coated specimens deposited at lower RF powers (20 W and 40 W, Figure 10b,c) exhibit minor surface perturbations with isolated sites of corrosion, yet without the extensive crack propagation observed at higher energy depositions, suggesting a relatively stable and corrosion‐resistant microstructure. The superior performance of the 80 W sample may be attributed to a balance between adatom mobility and film densification, resulting in a compact, homogenous coating morphology with minimal topographical irregularities, in alignment with classical thin film growth models such as Thornton's structure zone model. Conversely, the sample fabricated at 160 W (Figure 10e) exhibits pronounced corrosion‐induced damage, including widespread pitting and microcrack networks, indicative of structural vulnerability. These phenomena are closely associated with the increased surface roughness and topographical complexity previously quantified via AFM (Section 3.2), which align with the principles of surface energy minimization and the Wenzel wetting regime, wherein increased roughness amplifies the real contact area available for corrosive interaction. The abundance of asperities and irregular morphological features on the 160 W film provides multiple nucleation sites for corrosion, thereby undermining the integrity of the protective layer. This highlights a direct correlation between deposition parameters, morphological evolution, and electrochemical performance, wherein excessive RF power compromises coating uniformity, promoting defect‐assisted corrosion. Ultimately, the SEM data emphasize the pivotal role of surface architecture—namely roughness, porosity, and crack density—in dictating the corrosion resistance of CrN films, underscoring the necessity of optimizing RF power to engineer coatings with superior durability under aggressive environmental conditions.

## 4 Conclusions

This study explored the relationship between RF power levels and the resulting effects on the crystallinity, surface topography, and corrosion resistance of CrN thin films. The results revealed a clear correlation between increased RF power and improved crystallinity, with significant enhancements observed as the RF power rose from 20 W to a maximum of 160 W. XRD analysis confirmed the formation of more ordered CrN phases, with a preferential (111) orientation, as the RF power increased, signifying improved film quality and crystallinity. Increasing the RF power to its maximum of 80 W leads to a substantial enhancement in surface uniformity and a more evenly distributed grain pattern. However, beyond this threshold, an increase in surface roughness was observed, particularly for films deposited at 160 W, which may have contributed to the diminished corrosion resistance at higher RF powers. Utilizing Minkowski function diagrams for further examination revealed unique characteristics in the samples, especially in pinpointing singular voids which signify material properties at certain levels within the analyzed samples. This method offered a more profound understanding of the surface topology of the coated films. The corrosion resistance tests highlighted the crucial role of RF power in determining the protective capability of the CrN coatings. A distinct peak in corrosion resistance was found at 80 W, with further increases in RF power leading to a decline in performance. This suggests that while higher RF power contributes to improved crystallinity, it may also inadvertently lead to surface imperfections and roughness, which compromise the film's protective characteristics in corrosive environments. Overall, the findings of this study emphasize the critical influence of RF power on the physical and chemical properties of CrN thin films. By calibrating RF power precisely, it is possible to tailor the properties of CrN coatings to meet specific application requirements. This work provides valuable insights into the optimization of CrN thin films for a wide range of industrial applications, particularly in environments where both mechanical durability and corrosion resistance are paramount.

## Author Contributions

**Alireza Grayeli:** validation, writing – original draft, investigation, formal analysis, funding acquisition. **Sahar Rezaee:** writing – review and editing, investigation, software, data curation, project administration. **Ştefan Ţălu:** methodology, formal analysis, writing – review and editing, validation, visualization, resources, supervision, conceptualization.

## Conflicts of Interest

The authors declare no conflicts of interest.

## Data Availability

The data that support the findings of this study are available on request from the corresponding author. The data are not publicly available due to privacy or ethical restrictions.

## References

[jemt70059-bib-0001] Ahmadpourian, A. , C. Luna , A. Boochani , et al. 2016. “The Effects of Deposition Time on Surface Morphology, Structural, Electrical and Optical Properties of Sputtered ag‐Cu Thin Films.” European Physical Journal Plus 131: 1–7. 10.1140/epjp/i2016-16381-2.

[jemt70059-bib-0002] Aslam, R. , M. Mobin , S. Zehra , and J. Aslam . 2022. “A Comprehensive Review of Corrosion Inhibitors Employed to Mitigate Stainless Steel Corrosion in Different Environments.” Journal of Molecular Liquids 364: 119992. 10.1016/j.molliq.2022.119992.

[jemt70059-bib-0003] Babur, M. Z. , Z. Iqbal , M. Shafiq , M. Y. Naz , and M. M. Makhlouf . 2021. “Comparative Study of PVD Titanium Nitride Coating With Cathodic Cage Plasma Nitriding of Austenitic 201 Stainless Steel for Enhanced Tribological Properties.” Applied Physics A: Materials Science & Processing 127: 954. 10.1007/s00339-021-05109-0.

[jemt70059-bib-0004] Bastami, H. , A. Grayeli , A. Arman , et al. 2025. “Microstructure of ZrO_2_ Films: Hydrophilic Properties and Optical Band Gaps.” Materials Today Communications 45: 112338. 10.1016/j.mtcomm.2025.112338.

[jemt70059-bib-0005] Çeşmeli, S. , and C. Biray Avci . 2019. “Application of Titanium Dioxide (TiO_2_) Nanoparticles in Cancer Therapies.” Journal of Drug Targeting 27, no. 7: 762–766. 10.1080/1061186X.2018.1527338. 30252540

[jemt70059-bib-0006] Das, A. , S. Roychowdhury , and V. Kain . 2020. “Establishing the Passive Film Stability Formed at Different Depths From the Surface of Machined Type 304 L SS.” Corrosion Science 176: 109022. 10.1016/j.corsci.2020.109022.

[jemt70059-bib-0007] D'Souza, B. , A. Leong , Q. Yang , and J. Zhang . 2021. “Corrosion Behavior of Boronized Nickel‐Based Alloys in the Molten Chloride Salt.” Corrosion Science 182: 109285. 10.1016/j.corsci.2021.109285.

[jemt70059-bib-0008] Eidsvåg, H. , S. Bentouba , P. Vajeeston , S. Yohi , and D. Velauthapillai . 2021. “TiO_2_ as a Photocatalyst for Water Splitting: An Experimental and Theoretical Review.” Molecules 26, no. 6: 1687. 10.3390/molecules26061687. 33802911 PMC8002707

[jemt70059-bib-0009] Grayeli, A. , Ş. Ţălu , M. Bramowicz , et al. 2019. “Minkowski Functional Characterization and Fractal Analysis of Surfaces of Titanium Nitride Films.” Materials Research Express 6, no. 8: 086463. 10.1088/2053-1591/ab26be.

[jemt70059-bib-0010] Grudinin, V. A. , G. A. Bleykher , D. V. Sidelev , Y. N. Yuriev , and A. D. Lomygin . 2022. “Magnetron Deposition of Chromium Nitride Coatings Using a Hot Chromium Target: Influence of Magnetron Power on the Deposition Rate and Elemental Composition.” Surface and Coating Technology 433: 128120. 10.1016/j.surfcoat.2022.128120.

[jemt70059-bib-0011] Guo, S. , D. Xu , Y. Liang , et al. 2020. “Corrosion Characteristics of Typical Ni–Cr Alloys and Ni–Cr–Mo Alloys in Supercritical Water: A Review.” Industrial & Engineering Chemistry Research 59: 18727–18739. 10.1021/acs.iecr.0c04292.

[jemt70059-bib-0012] Gwyddion Software n.d. Accessed June 23, 2025. http://gwyddion.net/.

[jemt70059-bib-0013] Habibi, M. , S. Mirzaei , A. Arman , et al. 2022. “Microstructure, Fractal Geometry and Corrosion Properties of CrN Thin Films: The Effect of Shot Number and Angular Position.” Materials Today 32: 104072. 10.1016/j.mtcomm.2022.104072.

[jemt70059-bib-0014] Hoseinzadeh, T. , S. Solaymani , S. Kulesza , et al. 2018. “Microstructures, Fractal Geometry and Dye‐Sensitized Solar Cells Performance of CdS/TiO_2_ Nanostructures.” Electroanalytical Chemistry 830–831: 80–87. 10.1016/j.jelechem.2018.10.037.

[jemt70059-bib-0015] Huang, H. , D. Shiau , C. Chen , et al. 2019. “Nitrogen Plasma Immersion Ion Implantation Treatment to Enhance Corrosion Resistance, Bone Cell Growth, and Antibacterial Adhesion of Ti‐6Al‐4V Alloy in Dental Applications.” Surface and Coatings Technology 365: 179–188. 10.1016/j.surfcoat.2018.06.023.

[jemt70059-bib-0016] Karimi, M. , and A. R. Grayeli . 2019. “Improving the Corrosion Resistance of Ni/SS Thin Films by Nitrogen Ion Implantation.” Acta Physica Polonica A 16, no. 3: 536–541. 10.30509/pccc.2023.167128.1213.

[jemt70059-bib-0017] Knápek, A. , D. Sobola , D. Burda , A. Daňhel , M. Mousa , and V. Kolařík . 2019. “Polymer Graphite Pencil Lead as a Cheap Alternative for Classic Conductive SPM Probes.” Nanomaterials 9, no. 12: 1756. 10.3390/nano9121756. 31835524 PMC6956198

[jemt70059-bib-0018] Koseoglu, H. , F. Turkoglu , M. Kurt , et al. 2015. “Improvement of Optical and Electrical Properties of ITO Thin Films by Electro‐Annealing.” Vacuum 120, no. 8–13: 8–13. 10.1016/j.vacuum.2015.06.027.

[jemt70059-bib-0019] Kumar, C. , M. Shrivastav , J. Escrig , et al. 2024. “The Correlation Between Surface Scaling Behavior and Optical Properties of NiO Thin Films Nanostructures: An Investigation Based on Fractal Concept.” Ceramics International 50, no. 21: 41614–41627. 10.1016/j.ceramint.2024.08.011.

[jemt70059-bib-0020] Lei, M. , H. Z. Zhao , H. Yang , B. Song , and W. H. Tang . 2008. “Synthesis of Transition Metal Carbide Nanoparticles Through Melamine and Metal Oxides.” Journal of the European Ceramic Society 28: 1671–1677. 10.1016/j.jeurceramsoc.2007.11.013.

[jemt70059-bib-0021] Lim, K. G. , A. Handoko , S. Nemani , et al. 2020. “Rational Design of two‐Dimensional Transition Metal Carbide/Nitride (MXene) Hybrids and Nanocomposites for Catalytic Energy Storage and Conversion.” ACS Nano 14: 10834–10864. 10.1021/acsnano.0c05482. 32790329

[jemt70059-bib-0022] Liu, C. , Q. Bi , and A. Leyland . 2003. “An Electrochemical Impedance Spectroscopy Study of the Corrosion Behaviour of PVD Coated Steels in 0.5 N NaCl Aqueous Solution: Part II. EIS Interpretation of Corrosion Behaviour.” Corrosion Science 45: 1257–1273. 10.1016/S0010-938X(02)00214-7.

[jemt70059-bib-0023] Merie, V. V. , G. Negrea , and E. Modi . 2016. “The Influence of Substrate Temperature on the Tribo‐Mechanical Properties of Chromium Nitride Thin Films.” Materials Science and Engineering 147: 012020. 10.1088/1757-899X/147/1/012020.

[jemt70059-bib-0024] Nogueira, R. P. , J. D. Uchoa , F. Hilario , et al. 2021. “Characterization of Optimized TiO_2_ Nanotubes Morphology for Medical Implants: Biological Activity and Corrosion Resistance.” International Journal of Nanomedicine 16: 667–682. 10.2147/IJN.S285805. 33531806 PMC7847373

[jemt70059-bib-0025] Noorbakhsh, R. , S. Rezaee , B. Arghavani Nia , and A. Boochani . 2023. “Morphology and Multifractal Characteristics of ag–cu Films With N Doping Prepared by Direct Current Magnetron Sputtering Method.” Optical and Quantum Electronics 55, no. 10: 915. 10.1007/s11082-023-05158-0.

[jemt70059-bib-0026] Okonkwo, B. , C. Jeong , and C. Jang . 2022. “Advances on Cr and Ni Electrodeposition for Industrial Applications‐A Review.” Coating 12: 1555. 10.3390/coatings12101555.

[jemt70059-bib-0027] Ovcharenko, V. D. , A. S. Kuprin , G. N. Tolmachova , et al. 2015. “Deposition of Chromium Nitride Coatings Using Vacuum Arc Plasma in Increased Negative Substrate Bias Voltage.” Vacuum 117: 27–34. 10.1016/j.vacuum.2015.04.008.

[jemt70059-bib-0028] Pathak, S. , J. O. Morales‐Ferreiro , G. Silva‐Oelker , et al. 2025. “Insight Mechanisms of Complex Roughening Dynamics and Investigation of Fractal Parameters, Optical Constant, and Dispersion Parameters of CdS Thin Films for p‐n (n‐CdS/p‐Si) Heterojunction‐Based Photodetector.” Emergent Materials 8: 1–30. 10.1007/s42247-025-01018-7.

[jemt70059-bib-0029] Peri, R. , M. Bhagavathiachari , and S. Balasubramania . 2022. “A Detailed Study on the Electrochemical Properties of Transition Metal‐Based Carbide/Nitride Thin Films in Energy Conversion and Storage Devices.” Electrochimica Acta 427: 140860. 10.1016/j.electacta.2022.140860.

[jemt70059-bib-0030] Rezaee, S. , A. Arman , S. Jurecka , et al. 2020. “Effect of Annealing on the Micromorphology and Corrosion Properties of Ti/SS Thin Films.” Superlattices and Microstructures 146: 106681. 10.1016/j.apsusc.2011.08.014.

[jemt70059-bib-0031] Ruden‐Munoz, A. , E. Restrepo‐Parra , and F. Sequeda . 2015. “CrN Coatings Deposited by Magnetron Sputtering: Mechanical and Tribological Properties.” Dyna 82: 147–155. 10.15446/dyna.v82n191.43292.

[jemt70059-bib-0032] Sadeghi, M. , A. Arman , Ș. Ţălu , A. R. Grayeli , R. Shakoury , and A. Zelati . 2023. “Influence of Ion Implantation on Corrosion Resistance of the Nickel Over Steel.” Materials Science and Technology 39, no. 6: 660–670. 10.1080/02670836.2022.2131127.

[jemt70059-bib-0033] Shah, H. N. 2017. “Structural and Mechanical Characterisation of the Chromium Nitride Hard Coating Deposited on the Silicon and Glass Substrate.” International Journal of Automation and Mechanical Engineering 14: 3872–3886. 10.15282/ijame.14.1.2017.5.0315.

[jemt70059-bib-0034] Shakoury, R. , A. G. Korpi , K. Ghosh , et al. 2019. “Stereometric and Scaling Law Analysis of Surface Morphology of Stainless Steel Type AISI 304 Coated With Mn: A Conventional and Fractal Evaluation.” Materials Research Express 6: 116436. 10.1088/2053-1591/ab4aa6.

[jemt70059-bib-0035] Sobola, D. , S. Ramazanov , M. Konečný , et al. 2020. “Complementary SEM‐AFM of Swelling Bi‐Fe‐O Film on HOPG Substrate.” Materials 13: 2402. 10.3390/ma13102402. 32456133 PMC7287891

[jemt70059-bib-0036] Sobola, D. , Ş. Ţălu , S. Shahram , and G. Lubomír . 2017. “Influence of Scanning Rate on Quality of AFM Image: Study of Surface Statistical Metrics.” Microscopy Research and Technique 80, no. 12: 1328–1336. 10.1002/jemt.22945. 28905452

[jemt70059-bib-0037] Solaymani, S. , S. Kulesza , Ş. Ţălu , et al. 2018. “The Effect of Different Laser Irradiation on Rugometric and Microtopographic Features in Zirconia Ceramics: Study of Surface Statistical Metrics.” Journal of Alloys and Compounds 765: 180–185. 10.1016/j.jallcom.2018.06.213.

[jemt70059-bib-0038] Subramanian, B. , and M. Jayachandran . 2011. “Preparation of Chromium Oxynitride and Chromium Nitride Films by DC Reactive Magnetron Sputtering and Their Material Properties.” Corrosion Engineering Science and Technology 46: 554–561. 10.1179/147842209X12579401586807.

[jemt70059-bib-0039] Ţălu, Ș. 2015. Micro and Nanoscale Characterization of Three‐Dimensional Surfaces: Basics and Applications. Napoca Star Publishing House.

[jemt70059-bib-0040] Țălu, Ș. , R. P. Yadav , A. K. Mittal , et al. 2017. “Application of Mie Theory and Fractal Models to Determine the Optical and Surface Roughness of Ag‐Cu Thin Films.” Optical and Quantum Electronics 49: 256. 10.1007/s11082-017-1079-3.

[jemt70059-bib-0041] Vaca, L. S. , J. P. Quintana , D. Vega , A. Marquez , and S. P. Brühl . 2021. “Tribological and Corrosion Behavior of Duplex Coated AISI 316L Using Plasma Based Ion Implantation and Deposition.” Materials Today 26: 101892. 10.1016/j.mtcomm.2020.101892.

[jemt70059-bib-0042] Vinita, C. , R. P. Kumar , B. K. Yadav , and B. K. Singh . 2024a. “Impact of Surface‐Roughness and Fractality on Electrical Conductivity of SnS Thin Films.” Physica A: Statistical Mechanics and its Applications 654: 130165. 10.1016/j.physa.2024.130165.

[jemt70059-bib-0043] Vinita, C. , R. P. Kumar , B. K. Yadav , and B. K. Singh . 2024b. “Surfaces Properties Correlation With Optical Parameters of Thickness Dependent Self‐Affine Nanostructured SnS Thin Films: A Study Based on Scaling Law.” Colloids and Surfaces, A: Physicochemical and Engineering Aspects 691: 133865. 10.1016/j.colsurfa.2024.133865.

[jemt70059-bib-0044] Wang, Z. , Z. Feng , and L. Zhang . 2020. “Effect of High Temperature on the Corrosion Behavior and Passive Film Composition of 316 L Stainless Steel in High H2S‐Containing Environments.” Corrosion Science 174: 108844. 10.1016/j.corsci.2020.108844.

[jemt70059-bib-0045] Yan, L. T. , and J. K. Rath . 2011. “Electrical Properties of Vacuum‐Annealed Titanium‐Doped Indium Oxide Films.” Applied Surface Science 257, no. 22: 9461–9465. 10.1016/j.apsusc.2011.06.035.

[jemt70059-bib-0046] Zaffor, A. , F. Di Franco , and M. Santamaria . 2021. “Corrosion of Stainless Steel in Food and Pharmaceutical Industry.” Current Opinion in Electrochemistry 29: 100760. 10.1016/j.coelec.2021.100760.

[jemt70059-bib-0047] Zalnezhad, E. , A. Sarhan , and M. Hamdi . 2013. “Surface Hardness Prediction of CrN Thin Film Coating on Al7075‐T6 Alloy Using Fuzzy Logic System.” International Journal of Precision Engineering and Manufacturing 14: 467–473. 10.1007/s12541-013-0063-5.

